# Disparities in mental health care utilization among inpatients in various types of health institutions: a cross-sectional study based on EHR data in Shanghai, China

**DOI:** 10.1186/s12889-019-7343-7

**Published:** 2019-07-31

**Authors:** Jianwei Shi, Lan Tang, Limei Jing, Jinsong Geng, Rui Liu, Li Luo, Ning Chen, Qian Liu, Xin Gong, Xiaojie Bo, Yan Yang, Zhaoxin Wang

**Affiliations:** 1 School of Public Health, Shanghai Jiaotong University School of Medicine, Shanghai, 200025 China; 2 Yangpu Hospital, Tongji University School of Medicine, Shanghai, 200090 China; 3 Pudong Weifang Community Health Center, Shanghai, 200120 China; 4 Shanghai University of Traditional Chinese Medicine, Shanghai, 201203 China; 5 Department of Medical Informatics, Evidence-based Medical Center, Medical School of Nantong University, Nantong, 226001 China; 6 Shanghai Tenth People’s Hospital, Tongji University School of Medicine, Shanghai, 200072 China; 7 School of Public Health, Fudan University, Shanghai, 200032 China; 8 School of Medicine, Tongji University, Shanghai, 200092 China; 9 School of Economics and Management, Tongji University, Shanghai, 200092 China; 10 General Practice Center, Nanhai Hospital, Southern Medical University, 227 Chongqing South RD, Shanghai, 200025, 528244 Guangdong China

**Keywords:** Mental disorders, Utilization, Inpatient, Health policy, China

## Abstract

**Background:**

Reform of the health care system in China has prompted concerns about the utilization of mental health services. This study aims to compare the utilization of mental health services among inpatients in various types of health institutions in Shanghai (community health care centres, secondary general hospitals, tertiary general hospitals, and specialty hospitals).

**Methods:**

Based on electronic health record (EHR) data, we extracted all of the mental hospitalization data from various types of public health institutions in Pudong New Area, Shanghai, China, from 2013 to 2016. The distribution of mentally ill inpatients and the possible factors contributing to the observed differences in these institutions were analysed.

**Results:**

Specialty psychiatric hospitals in Pudong New Area, Shanghai, admitted more inpatients and treated in patients with more severe disorders (49.73%). However, those who were male (OR = 0.545), were elderly (OR = 20.133), had inferior insurance (urban social insurance for citizens: OR = 4.013; paying themselves, OR = 29.489), had a longer length of stay (OR = 1.001) and had lower costs (OR = 0.910) were more likely to choose community health centres than specialty hospitals. Those who preferred the secondary and tertiary hospitals to the specialty ones were more likely to be in the male, elderly, married, shorter length of stay and higher-cost groups. Notably, compared to those with urban social insurance for workers, those who had urban social insurance for citizens (OR = 3.136) or paid out-of-pocket (OR = 9.822) were significantly clustered in the tertiary hospitals rather than the specialty hospitals.

**Conclusions:**

Inpatients who were male, were older, had inferior insurance, had a longer length of stay and had lower costs preferred the elementary health services. However, the utilization of mental health care in high-tier institutions reflected defects, especially the fact that the current health insurance system does not adequately restrict patients’ choices, and those who paid more tended to choose tertiary hospitals instead of professional specialty ones. We suggest that psychiatric services should be enhanced by instituting reforms, including public education, improved health insurance, a forceful referral system, and competency reinforcement for primary care physicians, to provide a more integrated mental health system.

## Background

The Global Burden of Disease Study has recently reported the disease burden associated with mental, neurological, and substance use disorders, revealing that they represent a substantial burden worldwide [1, 2]. Notably, the analysis revealed that China accounted for the highest disease burden (17%) of global mental, neurological, and substance use disorders, with depressive disorders and anxiety disorders being the most common [2]. In China, statistical data from China’s Disease Prevention and Control Bureau showed that mental disorders accounted for 20% of the national disease burden in 2009, which far outweighed the burden of other major chronic diseases, such as cardiovascular and cerebrovascular diseases, respiratory system diseases, and cancer. The World Health Organization (WHO) estimates that this percentage of disease burden will rise to 25% in 2020 [3]. Moreover, according to a study by Xu (2016), the total costs of mental disorders in 2013 accounted for more than 15% of the total health expenditure in China [4].

However, research shows that only a small proportion of patients receive treatment [5, 6], and the actual incidence of mental disorders may be higher than what is reported in China [7], which also occurs in many other countries. Statistics from the WHO World Mental Health Survey (WMHS) showed that in the 26 countries participating in the survey, 35.50–50.30% of patients in developed countries did not seek treatment, while the percentages were between 76.30 and 85.40% in the participating developing countries. Even serious mental disorders that influence normal functioning were not treated at all [8]. In China, the situation is more serious. According to statistical data from the Ministry of Health, although the incidence of mental health disorders increased significantly from 1993 to 2013, the treatment rate showed a reverse trend [9]. In addition, a study based on a sample from the eastern and western regions of China in 2009 showed that the treatment rate was lower than 10%, and in the western province of Qinghai, it was only 2% [10].

The possible reasons for the low utilization of mental health services among the mentally ill include the stigma associated with mental illness and the impact it may have on employment opportunities and, thus, the family’s material circumstances and socioeconomic status, thereby compounding social inequity [7]. Since China has a conservative culture, people may be more reluctant to access mental health services. However, the extremely low utilization rate in China may be due to not only people’s unwillingness to receive mental health treatment but also the poor provision of mental health services [11, 12]. As revealed by 2010 national data from China’s Statistical Information Centre, the number of trained professionals available and their skills are grossly inadequate to meet the public’s needs. China has a much smaller mental health workforce ratio than that that in other upper-middle-income countries, and the range of services is much narrower. In addition, almost all of the mental health professionals in China work in public specialty psychiatric hospitals. In China, psychiatric services are provided primarily by public health institutions, including public psychiatric and general hospitals. As a category, general public hospitals comprise various levels of health institutions, including community health care institutions and secondary and tertiary hospitals. Higher-level hospitals are equipped with more advanced equipment, facilitating better health care information and provision. However, compared with those provided by specialized institutions, the mental health services provided by general public hospitals are usually not very comprehensive [13].

In recent years, a series of national reforms have been implemented in China with the goal of improving the quantity and quality of mental health services in public health institutions, especially for patients with severe mental disorders [14–19]. In 2009, related national guidelines were released by China’s Ministry of Health, including the “Guidelines for Management and Treatment of Severe Mental Disease” (2009) and “The National Basic Public Health Service Regulations” (2009) [15, 16]. Between 2010 and 2011, China made its initial large-scale national investment in the construction of mental health institutions, with 9.1 billion RMB going towards the reorganization and expansion of premises and 1.45 billion RMB towards the purchase of necessary equipment in 550 provincial, municipal, and prefectural mental health institutions. In addition, to improve workforce capability, the state invested 2.8 million RMB in a programme aiming to promote staff members’ capabilities [17]. In 2012, “China’s Mental Health Law” was implemented to establish the provided and required funding, access to care, and standards for mental health care [18]. According to the “National Mental Health Work Plan (2015-2020)” [19] released in 2015, the number of mental health professionals nationwide will be increased to 40,000 by 2020 in order to manage 80% of registered severe mental disorder patients, and 70% of counties will implement integrated approaches to mental health care (involving primary health, judicial, and public security agencies) and community rehabilitation services. Since the release of this report, greater importance has been placed on the construction of primary health care. Overall, the key aspect of these reforms was to establish a sound, integrated system for mental disorders in which mental health services are provided primarily by specialty psychiatric hospitals and assisted by psychiatric units in general secondary and tertiary hospitals, with primary general health institutions functioning as “gatekeepers” that mainly provide early detection, treatment and rehabilitation services for the public. However, the utilization of mental health services under the current health system is unclear, and few quantitative studies have examined this issue.

To fill the gap in the literature regarding the status of the utilization of mental health services in various health care institutions and its influencing factors under the current health care system, we focused on inpatient services, which are currently the target of reform for improving mental health services. We hypothesize that there may be differences between various institutions in severe mental health service utilization. We chose Shanghai because its economic and geographic development make it a good case study for China. At the end of 2016, Shanghai had a population of 14.40 million registered residents and 9.80 million non-registered residents, and its GDP per capita was the highest in China (113.6 thousand RMB) [20]. In terms of health reforms, Shanghai’s government always takes the lead, and the status of its mental health system reflects the cutting edge in China.

## Methods

### Study design and data collection

To quantitatively assess the utilization of mental health services, we obtained data with permission from the Information Center of the Health and Family Planning Commission of Pudong New Area, Shanghai. The EHR data of inpatients with mental health disorders in all public hospitals and health care centres were extracted from 2013 to 2016. We studied all of the institutions providing mental health services in Pudong New Area, Shanghai, the largest district in Shanghai with both urban and rural areas. From 2013 to 2016, it covered an average population of approximately 5.50 million (22% of the total population of Shanghai) [20]. The average life expectancy in Pudong New Area during this period was 83.19 years, and that for the whole population of Shanghai was 82.67 years. In addition, the per capita disposable incomes for Pudong New Area and Shanghai were 55,776 RMB and 54,305 RMB in 2016, respectively.

In our study, data were collected from all of the public health institutions in Pudong New Area during 2013–2016. In total, this region had 30 community health institutions (total = 42), 11 general secondary hospitals (total = 11), 4 general tertiary hospitals (total = 5), and 4 psychiatric specialty hospitals (total = 4) that admitted patients with mental disorders. All of these institutions were established by the government.

### Study subjects

The EHR systems of the health institutions in our study included the hospitalization information of inpatients who received their first diagnosis of a mental disorder between 2013 and 2016. In this study, mental disorder information for a total of 7,910 hospitalizations was stripped of identifying information and extracted from the EHR systems.

In most cities in China, EHRs have used a uniform two-part version since 2001. The first part contains the inpatients’ personal information, including their sex, age, identification card number, health insurance type, profession, and address. This information is usually provided by the patients or their families. The second part contains the inpatients’ hospitalization information, including their diagnosis code, discharge status, pathologic diagnosis (if available), and operation code (if relevant). This information is provided by the patient’s physician, which ensures its reliability. In terms of the diagnosis code, each inpatient is coded with an ICD-10 disease code by their physician. Therefore, we extracted the inpatients with mental disorders by using their ICD-10 codes [1]. Inpatients with ICD codes F00 to F99.999 were extracted.

### Statistical analysis

All data were analysed using SAS Software 9.30. Basic descriptive statistics were used to analyse the numbers and disease rankings of inpatients in the four types of health institutions. A Chi-square test (Cochran-Mantel-Haenszel test) and one-way ANOVA were used to examine the inpatients’ demographics (sex, age, residence status, marital status), health insurance (namely, urban social insurance for workers, urban social insurance for citizens, new rural cooperative medical insurance, self-paying or other; the reimbursement ratio showed a decreasing tendency in that order. For instance, in Shanghai, the reimbursement ratio for inpatients with urban social insurance for workers was 85% and was 92% for employees and retired in all types of health institutions; for inpatients with urban social insurance for citizens, the reimbursement ratios were between 75 and 85% in the community health institutions, 65–75% in the secondary hospitals, and 55–65% in the tertiary hospitals, as well as 55–85% in the specialized institutions; for inpatients with new rural cooperative medical insurance, the reimbursement ratios were 60% in the community health institutions, 40% in the secondary, and 30% in the tertiary, as well as 30–40% in the specialized institutions), and hospital resource use (length of stay, inpatient total cost, out-of-pocket cost) among the various types of health institutions was examined to reveal the utilization characteristics. Importantly, to determine the associations between health institution type and possible influencing factors, we used multivariate logistic regression.

## Results

### Comparison of inpatients’ utilization of mental health services across various types of health care institutions

**Fig. 1:**
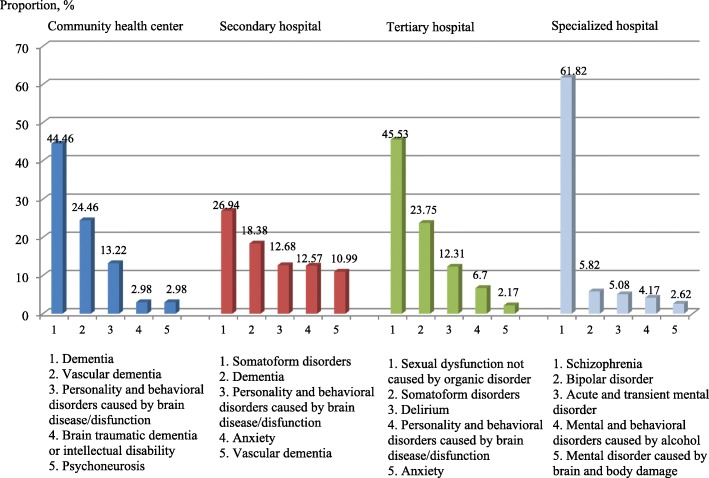
Rankings of the top mental health disorders in various types of public health institutions

A total of 7,910 hospitalizations of mental inpatients from 50 public health institutions in Pudong New Area during 2013–2016 were included, which were distributed across the four types of public institutions (community health centres, general secondary hospitals, general tertiary hospitals and specialty hospitals). In addition, statistics showed that mentally ill patients accounted for 6.37% (7,910/1,242,728) of the total chronic disease hospitalizations in these hospitals during this period. The numbers of mentally ill individuals in the four types of public health institutions are presented in Table 1. Most of them chose specialty hospitals (3,934/7,910, 49.73%), followed by secondary hospitals (1,893/7,910, 23.93%), tertiary hospitals (1,478/7,910, 18.69%), and community health centres (605/7,910, 7.65%). The data indicated that only two of the four specialty hospitals recruited enrolled more than 1,000 inpatients during the four-year period. However, the other two specialty hospitals admitted only 139 inpatients total during this time. Two of the total four tertiary hospitals in this area had between 477 and 910 inpatients. Of the 11 secondary general hospitals, only three had between 200 and 571 inpatients. Among the primary health care centres, the data showed that all 30 institutions had a sample size smaller than 200; 26 of these institutions admitted fewer than 50 inpatients from 2013 to 2016. The rankings and proportions of the top five mental disorders for each hospital type are presented in Fig. 1. Specialty hospitals experienced variation in the percentage of mental disorders, with schizophrenia being the highest (61.82%), followed by bipolar disorder (5.82%), acute and transient mental disorders (5.08%), mental and behavioural disorders caused by alcohol (4.17%), and anxiety (2.62%). In the general health institutions, dementia, somatoform disorders, and personality and behavioural disorders caused by brain disease/dysfunction were ranked as the top conditions. The mental disorders in specialty hospitals were more severe than those in the other hospital types. In addition, inpatients in community health centres had a similar disease spectrum to those in secondary and tertiary hospitals, but anxiety, which was less severe, was more common in the higher-tier hospitals.

**Table 1:** Distribution of mental health hospitalizations in various types of health institutions from 2013 to 2016

Variable	Community health centre *N* = 605	Secondary general hospital *N* = 1893	Tertiary general hospital *N* = 1478	Specialty hospital *N* = 3934	Total/average *N* = 7910
Number of health institutions with various hospitalization size, n(%)					
> 1000	0 (0.00)	0 (0.00)	0 (0.00)	2 (50.00)	2 (4.00)
501–1000	0 (0.00)	1 (8.33)	1 (25.00)	0 (0.00)	2 (4.00)
201–500	0 (0.00)	2 (16.67)	1 (25.00)	0 (0.00)	3 (6.00)
101–200	2 (6.67)	2 (16.67)	0 (0.00)	0 (0.00)	4 (8.00)
51–100	2 (6.67)	4 (33.33)	1 (25.00)	2 (50.00)	9 (18.00)
0–50	26 (86.67)	3 (25.00)	1 (25.00)	0 (0.00)	30 (60.00)
Total	30 (100.00)	12 (100.00)	4 (100.00)	4 (100.00)	50 (100.00)
Number of inpatients in various hospitalization groups, n(%)					
> 1000	0 (0.00)	0 (0.00)	0 (0.00)	3795 (96.47)	3795 (47.98)
501–1000	0 (0.00)	571 (30.16)	910 (61.57)	0 (0.00)	1481 (18.72)
201–500	0 (0.00)	762 (40.25)	477 (32.27)	0 (0.00)	1239 (15.66)
101–200	203 (33.55)	293 (15.48)	0 (0.00)	0 (0.00)	496 (6.27)
51–100	148 (24.46)	243 (12.84)	75 (5.07)	139 (3.53)	605 (7.65)
0–50	254 (41.98)	24 (1.27)	16 (1.08)	0 (0.00)	294 (3.72)
Total	605 (100.00)	1893 (100.00)	1478 (100.00)	3934 (100.00)	7910 (100.00)

### Comparison of inpatients’ characteristics and hospital resource use across various types of health care institutions

Table 2 shows the inpatients’ personal characteristics and hospital resource utilization in various types of public health institutions from 2013 to 2016. With regard to the sex of the inpatients, the results indicated that males were more inclined to choose tertiary hospitals (male/female: 70.77%/29.23%) and specialty hospitals (male/female: 56.61%/43.39%). Tertiary hospitals and specialty hospitals showed a relatively uniform distribution across age intervals, indicating that they attracted a wide range of inpatients. Community health centres (75.04%) and secondary hospitals (41.94%) showed higher rates of inpatients 70 years of age and older than tertiary hospitals (10.08%) and specialty hospitals (3.86%). However, the younger group showed the reverse trend. With respect to residence status, there were more non-registered residents in tertiary hospitals (41.47%) than in other hospitals. Unmarried (40.81%) and divorced inpatients (9.36%) were more prevalent in specialty hospitals than in general institutions (*p* < 0.001). Interestingly, the statistics showed that most patients in tertiary hospitals paid out-of-pocket (61.10%). In addition, the results showed that the average rate of hospitalizations per bed was highest in the tertiary hospitals (99.89, 0.78%) and lowest in the community health centres (93.99, 12.16%). However, the average length of stay was only 6.01 days in tertiary hospitals. The statistics also showed that inpatients in specialty hospitals (240.58 days) and community health centres (95.51 days) had longer lengths of stay. Correspondingly, inpatients in specialty hospitals paid the most for care (33.04 thousand RMB). The results also indicated that the out-of-pocket costs for inpatients in specialty hospitals were the lowest considering the margin (3.04/33.04). The average total cost (5.68 thousand RMB) and out-of-pocket cost (0.89 thousand RMB) were lowest in tertiary hospitals.

**Table 2:** Distribution of exposure to the four types of institutions by demographics and hospital resource use from 2013 to 2016, n(%)

Variable	Community health centre	Secondary general hospital	Tertiary general hospital	Specialty hospital	Total	*P*-value
	(n = 605)	(n = 1893)	(n = 1478)	(n = 3934)	(n = 7910)	
Personal demographics						
Sex						
Male	175 (28.93)	842 (44.48)	1046 (70.77)	2227 (56.61)	4290 (54.24)	< 0.001
Female	430 (71.07)	1051 (55.52)	432 (29.23)	1707 (43.39)	3620 (45.76)	
Age (year)						
≤ 30	1 (0.17)	72 (3.80)	346 (23.41)	964 (24.50)	1383 (17.48)	< 0.001
31–50	33 (5.45)	237 (12.52)	546 (36.94)	1681 (42.73)	2497 (31.57)	
51–70	117 (19.34)	790 (41.73)	437 (29.57)	1137 (28.90)	2481 (31.37)	
> 70	454 (75.04)	794 (41.94)	149 (10.08)	152 (3.86)	1549 (19.58)	
Residence status						
Non-registered resident	2 (0.33)	118 (6.23)	613 (41.47)	703 (17.87)	1436 (18.15)	< 0.001
Registered resident	603 (99.67)	1775 (93.77)	865 (58.53)	3231 (82.13)	6474 (81.85)	
Marital status (missing = 20)						
Unmarried	19 (3.15)	96 (5.12)	167 (11.30)	1605 (40.81)	1887 (23.92)	< 0.001
Married	347 (57.45)	1674 (89.28)	1303 (88.16)	1497 (38.06)	4821 (61.10)	
Widowed	210 (34.77)	81 (4.32)	3 (0.20)	113 (2.87)	407 (5.16)	
Divorced	11 (1.82)	14 (0.75)	3 (0.20)	368 (9.36)	396 (5.02)	
Other	17 (2.81)	10 (0.53)	2 (0.14)	350 (8.90)	379 (4.80)	
Health insurance						
Urban social insurance for workers	196 (32.40)	1402 (74.06)	463 (31.33)	1941 (49.34)	4002 (50.59)	< 0.001
Urban social insurance for citizens	165 (27.27)	111 (5.86)	97 (6.56)	689 (17.51)	1062 (13.43)	
New rural cooperative medical Insurance	62 (10.25)	37 (1.95)	7 (0.47)	213 (5.41)	319 (4.03)	
Self-pay	170 (28.10)	281 (14.84)	903 (61.10)	1020 (25.93)	2374 (30.01)	
Other	12 (1.98)	62 (3.28)	8 (0.54)	71 (1.80)	153 (1.93)	
Hospital resource use						
Hospitalizations per bed, %, M (SD)	94.23 (2.21)	96.60 (4.39)	99.89 (0.78)	97.44 (4.80)	94.23 (2.21)	< 0.001
Length of stay, days, M (SD)	95.51 (183.80)	29.64 (50.81)	6.01 (10.71)	240.58 (766.92)	135.17 (554.19)	< 0.001
Total cost, thousand RMB, M (SD)	7.82 (7.53)	17.96 (26.57)	5.68 (13.37)	33.04 (67.53)	22.39 (51.03)	< 0.001
Out-of-pocket cost, thousand RMB, M (SD)	6.73 (11.21)	4.49 (13.21)	0.89 (2.80)	3.04 (12.26)	3.11 (11.34)	< 0.001

Note: *P*-values based on a Chi-square test for categorical measures and ANOVA for continuous measures

### Multiple logistic analysis of mental health services utilization across various types of health care institutions

Multiple logistic regression was used to analyse the differences in inpatients’ utilization of services at various health care institutions (Table 3). Inpatients’ choice of specialty hospital was set as the reference. A comparison of community health centres and specialty hospitals indicated that inpatients from the former were significantly less likely to be female (OR = 0.545, 95% CI: 0.331–0.898, *p* = 0.017), and the comparisons were more significant between secondary and specialty hospitals (OR = 0.345, 95% CI: 0.259–0.459, *p* < 0.0001) and between tertiary and specialty hospitals (OR = 0.196, 95% CI: 0.146–0.264, p < 0.0001). There was an increasing trend with age of inpatients going to community health centres rather than specialty hospitals. In terms of insurance, inpatients with urban social insurance for citizens (OR = 4.013, 95% CI: 1.995–8.072, *p* < 0.001) and self-paying inpatients (OR = 29.489, 95% CI: 16.161–53.810, p < 0.001) were more likely to choose community health centres than those with urban social insurance for workers. Regarding hospital resource use, inpatients with percentage of hospitalization per bed were more likely to choose specialty hospitals than other health institutions. Inpatients with a higher average length of stay had a significantly higher likelihood of choosing community health centres (OR = 1.001, 95% CI: 1.001–1.001, *p* < 0.001). However, as the cost increased, inpatients showed a tendency to choose specialty hospitals (OR = 0.910, 95% CI: 0.879–0.941, *p* < 0.001), while more chose community health centres when the out-of-pocket cost increased (OR = 1.025, 95% CI: 1.016–1.035, *p* < 0.001).

**Table 3:** Logistic analysis of the utilization of mental health services in various health care institutions

Variable	Community health centre VS Specialty hospital			Secondary hospital VS Specialty hospital			Tertiary hospital VS Specialty hospital		
	OR	95% CI	*P*	OR	95% CI	*P*	OR	95% CI	*P*
Sex									
Male	1.000	Reference		1.000	Reference		1.000	Reference	
Female	0.545	0.331–0.898	0.017	0.345	0.259–0.459	<.0001	0.196	0.146–0.264	<.0001
Age									
≤30	1.000	Reference		1.000	Reference		1.000	Reference	
31–50	20.133	2.194–184.729	0.008	2.637	1.573–4.422	0.0002	0.883	0.587–1.329	0.550
51–70	–	–	–	7.967	4.643–13.670	< 0.001	1.340	0.836–2.147	0.224
> 70	–	–	–	86.600	44.542–168.370	< 0.001	7.654	4.116–14.235	<.0001
Residence status									
Non-registered resident	1.000	Reference		1.000	Reference		1.000	Reference	
Registered resident	–	–	–	0.487	0.329–0.720	0.0003	0.332	0.237–0.467	<.0001
Marital status (missing = 20)									
Unmarried	1.000	Reference		1.000	Reference		1.000	Reference	
Married	0.899	0.409–1.977	0.791	8.302	5.276–13.063	<.0001	14.945	10.031–22.266	<.0001
Widowed	1.478	0.496–4.406	0.484	2.681	1.112–6.464	0.028	1.346	0.320–5.664	0.685
Divorced	0.885	0.145–5.421	0.895	0.629	0.188–2.105	0.452	0.459	0.122–1.726	0.249
Other	0.335	0.058–1.923	0.220	0.362	0.115–1.146	0.084	0.093	0.019–0.455	0.003
Health insurance									
Urban social insurance for workers	1.000	Reference		1.000	Reference		1.000	Reference	
Urban social insurance for citizens	4.013	1.995–8.072	<.0001	0.997	0.593–1.679	0.992	3.136	1.833–5.366	<.0001
New rural cooperative medical insurance	1.743	0.468–6.493	0.408	0.419	0.154–1.141	0.089	0.160	0.049–0.527	0.003
Self-pay	29.489	16.161–53.810	<.0001	3.486	2.457–4.945	<.0001	9.822	7.037–13.709	<.0001
Other	4.355	0.757–25.039	0.099	4.807	1.196–19.309	0.027	1.538	0.314–7.548	0.596
Hospital resource use									
Hospitalizations per bed, %	0.353	0.323–0.386	<.0001	0.390	0.360–0.423	<.0001	0.437	0.403–0.475	<.0001
Length of stay, days	1.001	1.000–1.001	<.0001	0.947	0.941–0.952	<.0001	0.883	0.873–0.893	<.0001
Total cost, thousand RMB	0.910	0.879–0.941	<.0001	1.181	1.154–1.209	<.0001	1.198	1.169–1.228	<.0001
Out-of-pocket cost, thousand RMB	1.025	1.016–1.035	<.0001	0.988	0.975–1.001	0.064	0.843	0.818–0.870	<.0001

Note: --: The OR is ineffective because the frequency of this group in the community health centre is too low

Males (OR = 0.345, 95% CI: 0.259–0.459, p < 0.001) and younger inpatients had a higher probability of visiting secondary hospitals rather than specialty hospitals. In this study, the registered residents preferred specialty hospitals to secondary ones (OR = 0.487, 95% CI: 0.329–0.720, *p* = 0.0003). Significantly more married than unmarried inpatients chose secondary hospitals (OR = 8.302, 95% CI: 5.276–13.063, *p* < 0.001). Compared to those with social insurance for workers, inpatients who paid out-of-pocket significantly preferred secondary hospitals (OR = 3.486, 95% CI: 2.457–4.945, *p* < 0.001). In addition, inpatients with longer lengths of stay (OR = 0.947, 95% CI: 0.941–0.952, *p* < 0.001) and lower costs (OR = 1.181, 95% CI: 1.154–1.209, *p* < 0.001) were more likely to choose specialty hospitals than secondary hospitals. However, the out-of-pocket costs did not influence their choice of institutions (*p* = 0.064).

A comparison of tertiary hospitals with specialty hospitals showed that fewer females (OR = 0.196, 95% CI: 0.146–0.264, *p* < 0.001) chose the former. There were significantly more inpatients in the > 70 years age group than in the < 30 years age group (OR = 7.654, 95% CI: 4.116–14.235, *p* < 0.001). More registered than non-registered residents chose specialty hospitals rather than tertiary ones (OR = 0.332, 95% CI: 0.237–0.467, *p* < 0.001). Married individuals preferred tertiary hospitals more than unmarried individuals (OR = 14.945, 95% CI: 10.031–22.266, p < 0.001). Regarding insurance, more inpatients with social insurance for citizens (OR = 3.136, 95% CI: 1.833–5.366, p < 0.001) and who paid themselves (OR = 9.822, 95% CI: 7.037–13.709, p < 0.001) chose tertiary hospitals, but the situation was different for those with new rural cooperative medical insurance (OR = 0.160, 95% CI: 0.049–0.527, *p* = 0.003). In addition, inpatients with longer average lengths of stay (OR = 0.883, 95% CI: 0.873–0.893, p < 0.001) and higher out-of-pocket costs (OR = 0.843, 95% CI: 0.818–0.870, p < 0.001) were less likely to choose tertiary hospitals. However, more inpatients used tertiary hospitals as the total cost increased (OR = 1.198, 95% CI: 1.169–1.228, p < 0.001).

## Discussion

In this study, we examined the integrity of China’s mental health system by analysing inpatients’ utilization of mental health services in various types of health institutions in Shanghai and found that the current utilization of mental health service was not sound. The results showed that many of the community health centres also admitted inpatients, but most admitted very small numbers, indicating that they were not as competitive when providing services for severe cases. Interestingly, a large proportion of those who utilized the inpatient services of community health centres were elderly, had inferior health insurance and paid the least out-of-pocket considering their length of stay. This trend likely occurs because the elderly have complicated chronic diseases and require long-term care, and the community health centre is a better choice for them given its cost-effectiveness [21, 22]. For inpatients with inferior health insurance, the cost of service at community health centres is lower, which this group finds attractive [22]. However, there may be several reasons for most inpatients’ unwillingness to choose community health centres: (1) although policies have aimed at enhancing the service capacity of basic health institutions, due to their large perceived gap with the high-tier hospitals, manifested in an inferior workforce and facilities, inpatients do not prefer them. As revealed in He’s (2014) study, the initial diagnosis and recovery rates of mental health disorders for primary care physicians are only 31.25 and 27.17%, respectively [23]. (2) This problem is reinforced by the poor referral system in China. Currently, the health system aims to direct people with common diseases to elementary health care units and those with severe diseases to be transferred to secondary and tertiary hospitals, as well as specialized hospitals. However, because of the lack of an effective referral policy, the public can freely choose any kind of hospital [24, 25]. In contrast to this system, the collaborative care model, in which primary care physicians and mental health professionals work together to provide better service to patients, has been adopted in many developed countries or regions. Primary care physicians can obtain assistance from specialists and gradually improve their skills and win patients’ trust [26, 27].

Consistent with China’s “Guidelines for Management and Treatment of Severe Mental Disease” (2009) and “The National Basic Public Health Service Regulation” (2009), as well as the “National mental health work plan” (2015–2020), which indicate that specialized psychiatric hospitals should take primary responsibility for treating inpatients with mental disorders [15, 16, 19], our study confirmed that most inpatients who were clustered in this type of institution had more severe illness. Those who were female, younger, registered residents, and holders of urban social insurance for workers or new rural cooperative medical insurance preferred specialty hospitals, though such institutions may be associated with a longer length of stay than general public hospitals. These inpatients’ preference for specialty hospitals can be attributed primarily to the severity of their illness and their trust in the specialized nature of specialty hospitals’ care. In this study, we confirmed that inpatients with more serious and acute mental disorders, including schizophrenia, bipolar disorder, and acute and transient mental disorders, were primarily admitted to specialty institutions. However, the data showed that during the four-year study period, two of the four specialized hospitals admitted only 139 inpatients, which was far lower than the number admitted to some of the general hospitals. These specialized hospitals are likely on a smaller scale, and their service provision may not be as attractive, as has been stated and certified in other studies in China. In Liu et al. (2013)‘s study, which was conducted with data obtained from the Statistical Information Centre of the Ministry of Health in 2010, 29% of the psychiatrists from the 757 psychiatric hospitals nationwide had only a technical school degree, and 14% of them had no academic degree at all. Among the nurse group, 46% had no academic qualifications [13]. Thus, even many specialized hospitals may lack a qualified workforce. To improve the service abilities of professionals and nurses, effective training should be developed and provided to the staff of these specialized institutions.

Although the total number of mentally ill patients was higher in specialized hospitals, the large numbers of inpatients in secondary and tertiary public hospitals in China during this period also warrant attention. One possible reason for the high volume of severe mental health services utilization in these hospitals may be that secondary and tertiary public hospitals in China have been considered primary health service providers ever since the establishment of the health delivery system in the 1950s. The Chinese public is more inclined to rely on higher-tier, more sophisticated public general hospitals since these hospitals are thought to have a more advanced workforce and facilities [28–30]. In addition, the deficient referral system cannot reasonably guide and restrict patients’ behaviour. In this study, we found that although health insurance type can restrict inpatients’ choice of community health care centres, it cannot influence patients’ access to higher-tier hospitals. For instance, non-registered residents, who had inferior health insurance compared with registered residents, were more inclined to choose tertiary hospitals instead of other hospitals, and as the hospital tier increased, more of them paid out-of-pocket or with other inferior insurance. The non-registered residents were faced with structural constraints in terms of the health insurance system. We also found that the registered residents’ tendency to use specialty hospitals was not related to the type of insurance since there were significant relationships with both urban social insurance for workers (with the highest reimbursement ratio) and new rural cooperative medical insurance (with the lowest reimbursement ratio). It was also found that inpatients with higher total costs and lower out-of-pocket cost ratios were clustered in tertiary hospitals, but overall, they had less serious mental diseases than inpatients at specialty hospitals. To avoid improper hospitalization in tertiary hospitals, according to the health department, strict restrictions on the length of stay for these patients were implemented in the 2000s, which is why we found that patients had a shorter length of stay in tertiary hospitals. The existing problem suggests that good public education on the functions of various hospitals should be widely and thoroughly provided. Moreover, to improve the health insurance system and better guide the public’s behaviour, government financing for health care should be undertaken using a supply-side financing model, where government financial support goes directly to health care providers, in contrast to the current demand-side model, where it is provided through medical insurance or subsidies [31]. Most importantly, a forceful referral system should be created and implemented to restrict patients’ behaviours.

There were several notable limitations in this study. First, as the sample was chosen from Pudong New Area in Shanghai, China, the survey may not be representative of other areas, especially underdeveloped ones. The study should be extended to include a larger sample of mental health institutions in a greater number of regions. Second, we examined the integrity of the mental health system from the inpatient perspective; it would be better to combine this analysis with an analysis from the institutional perspective, including the assessment of their facilities and workforce. Third, in this study, only public institutions were included because in China, private mental institutions currently account for a very small proportion and are not comparable to public ones.

## Conclusions

Despite these limitations, the findings from this study are helpful for informing policy decisions and practices to improve the mental health system. Our study found that although China has made great efforts to improve the mental health system for many years, inpatients’ utilization of services still reflects many defects. In addition to personal characteristics, these defects were also caused by the improper health policy atmosphere, including the insufficient workforce, health insurance restrictions, and referral system. Thus, specialty hospitals should further improve their capabilities. Public education, the implementation of policies on provider-oriented health insurance, a forceful referral system and competency reinforcement for primary care physicians should also be undertaken.

## Data Availability

The datasets generated and/or analysed during the current study are available in the Figshare repository (https://figshare.com/s/7703654c81e4f41ccf5f).

## Abbreviations

EHR: Electronic Health Record
RMB: Renminbi, or Chinese currency

## References

[1] Murray CJ, Vos T, Lozano R, Naghavi M, Flaxman AD, Michaud C (2010). Disability-adjusted life years (DALYs) for 291 diseases and injuries in 21 regions, 1990-2010: a systematic analysis for the global burden of disease study. Lancet..

[2] Charlson FJ, Baxter AJ, Cheng HG, Shidhaye R, Whiteford HA (2016). The burden of mental, neurological, and substance use disorders in China and India: a systematic analysis of community representative epidemiological studies. Lancet..

[3] Projections of mortality and burden of disease to 2030. Geneva: World Health Organization. 2006. http://www.who.int/healthinfo/statistics/bodprojections2030/en/index.html. Accessed 10 Nov 2017.

[4] Xu J, Wang J, Wimo A, Qiu CX (2016). The economic burden of mental disorders in China, 2005-2013: implications for health policy. BMC Psychiatry.

[5] Mathias K, Kermode M, San Sebastian M, Koschorke M, Goicolea I (2015). Under the banyan tree-exclusion and inclusion of people with mental disorders in rural North India. BMC Public Health.

[6] Murthy RS (2011). Mental health initiatives in India (1947-2010). Natl Med J India.

[7] Liu S, Page A (2016). Reforming mental health in China and India. Lancet..

[8] WHO World Mental Health Survey Consortium (2004). Prevalence, severity, and unmet need for treatment of mental disorders in the World Health Organization world mental health surveys. JAMA..

[9] National Health and Family Planning Commission of the People's Republic of China. China health statistics yearbook. http://www.nhfpc.gov.cn/zwgkzt/tjnj/list.shtml. Accessed 10 Nov 2017.

[10] Philips MR, Zhang J, Shi Q, Song Z, Pang S, Zhang Y (2009). Prevalence, treatment, and associated disability of mental disorders in four provinces in China during 2001-05: an epidemiological survey. Lancet..

[11] Huang C, Yu H, Koplan JP (2014). Can China diminish its burden of non-communicable diseases and injuries by promoting health in its policies, practices, and incentives?. Lancet..

[12] Liu J, Ma H, He YL, Xie B, Xu YF, Tang HY (2011). Mental health system in China: history, recent service reform and future challenges. World Psychiatry.

[13] Liu CP, Chen LJ, Xie B, Yan J, Jin TL, Wu ZG (2013). Number and characteristics of medical professionals working in Chinese mental health facilities. Shanghai Arch Psychiat (Chin J).

[14] Chen WF (2009). Study on China's development of community mental health services and countermeasures.

[15] China's Ministry of Health (2009). Notification on issuing guides for management and treatment of severe mental disease.

[16] China's Ministry of Health (2009). The National Basic Public Health Service Regulation.

[17] Sun YF, Chen YX, Hui W, Wu HZ (2012). Research of beds allocating and planning of mental health institutions in China. Chin Hospital Manage (Chin J).

[18] The Central People's Government of the People's Republic of China (2012). China's mental health law.

[19] The State Council of China (2015). National mental health work plan (2015–2020).

[20] Shanghai Statistics Bureau (2017). Statistical bulletin of Shanghai's national economic and social development (2016).

[21] Liu X, Zhang XJ, Wang HY. Survey on the chronic disease incidence and demand of community health services of the old. Chin Health Indus (Chinese J). 2013;12(2):77–8.

[22] Liu Y, Zhai CK, Lu Y, Sun GJ, Zhang H, Zhang HF (2014). Survey of chronic diseases, health related behaviors, and current health demands of Nanjing residents. J Shanghai Jiaotong Univ Med Sci (Chin J).

[23] He XD (2014). The prevalence of mental disorders and its diagnosis and treatment of mental disorders in the community hospitals. Bio Tech World.

[24] Wang Z, Shi J, Wu Z, Xie H, Yu Y, Li P (2017). Changes in chronic disease management among community health centers (CHCs) in China: has health reform improved CHC ability?. Int J Health Plann Manag.

[25] Zhao L, Zhang Y, Hou YB, Yan GM (2018). Efficiency of community health centers in China during 2013-2015: A synchronic and diachronic study. Family Med Commun Health.

[26] Bartels SJ, Coakley EH, Zubritsky C, Ware JH, Miles KM, Areán PA (2004). Improving access to geriatric mental health services: a randomized trial comparing treatment engagement with integrated versus enhanced referral care for depression, anxiety, and at-risk alcohol use. Am J Psychiatry.

[27] Gilbody S, Bower P, Fletcher J, Richards D, Sutton AJ (2006). Collaborative care for depression: a cumulative meta-analysis and review of longer-term outcomes. Arch Intern Med.

[28] Liu GG, Vortherms SA, Hong XZ (2017). China's health reform update. Annu Rev Public Health.

[29] Tong HL, Jiao XN, Gu XB, Wen Q, Men ZH, Liu CT (2015). Awareness of mental health-related knowledge and attitude towards patients with mental disorders among community residents in Harbin city and the influencing factors. Pract Prevent Med (Chin Jl).

[30] Deng ZJ (2016). Research on the mental inpatients' stigma and self-identification.

[31] Liu GG, Krumholz S. Economics of health transitions in China. In: Fan S, Kanbur R, Wei S-J, Zhang X, editors. The Oxford companion to the economics of China. Oxford: Oxford Univ. Press; 2014. p. 449–55.

